# GPS tracking reveals landfill closures induce higher foraging effort and habitat switching in gulls

**DOI:** 10.1186/s40462-021-00278-2

**Published:** 2021-11-12

**Authors:** Liam P. Langley, Stuart Bearhop, Niall H.K. Burton, Alex N. Banks, Tim Frayling, Chris B. Thaxter, Gary D. Clewley, Emily Scragg, Stephen C. Votier

**Affiliations:** 1 Centre for Ecology and Conservation, University of Exeter, Penryn, TR10 9EZ UK; 2 British Trust for Ornithology, The Nunnery, Thetford, IP24 2PU UK; 3 Natural England, Sterling House, Exeter, EX1 1QA UK; 4 Lyell Centre, Heriot-Watt University, Edinburgh, UK

**Keywords:** PAFS, Anthropogenic Change, Lesser Black-backed Gull, *Larus fuscus*, Generalists, Management

## Abstract

**Background:**

Landfills are a major subsidy for some animals, with implications for their life history and demography. Gulls feed extensively on food from landfills and closures are expected to have ecological consequences, but how this influences movement ecology is virtually unknown.

**Methods:**

We used GPS-tracking to quantify foraging behaviour and habitat choice of lesser black-backed gulls (*Larus fuscus*) breeding at two colonies before and after closure of two nearby landfills.

**Results:**

Following closure, gulls from both colonies travelled further and for longer to forage. Gulls also changed habitat selection, although this differed by colony - birds from one colony shifted to agricultural habitats, while at the other, increased their use of urban areas. These behavioural responses had no effect on adult body condition but hint at potential direct effects of higher foraging costs and indirect impacts by shifting to new habitats.

**Conclusions:**

Our results demonstrate how landfill availability influences gull foraging movements and habitat selection. We also emphasize the value of biologging to detect rapid behavioural responses in contrast to more conventional demographic approaches, which is especially important for animals that spend the majority of their lives away from direct observation.

**Supplementary Information:**

The online version contains supplementary material available at 10.1186/s40462-021-00278-2.

## Introduction

Human activities have negatively impacted biodiversity [1, 2], but can also provide ecological opportunities including the provision of predictable anthropogenic food subsidies (hereafter PAFS; [3]). Given the large number of species and individuals that feed on PAFS [3], understanding the ecological consequences of changes in the availability of subsidies such as landfill refuse is imperative.

Global solid urban waste production in 2000 was ~ 3 million tonnes per day [4], which includes large amounts of food dumped into landfills [4, 5]. This provides nourishment for large numbers of animals, predominantly opportunistic birds and mammals [6]. However, while global waste production is projected to peak next century [4], there is currently an increased emphasis on sustainability and changing waste management in developed nations, such as landfill closure (e.g. EU Directive 2018/850). These changes will greatly reduce food availability for landfill foragers, with poorly known ecological consequences [3].

Gulls (*Laridae*) forage regularly at landfills around the world [7–16]. As such, landfills have underpinned 20th Century gull population increases [13, 17, 18] and range expansions [11, 19–21]. However, ongoing changes in waste management, including landfill closures, are reducing the availability of this key resource. These changes are likely to directly impact gull demography [22, 23], although implications for their movement ecology are unknown.

Monitoring the effects of landfill closures on gulls normally focuses on demography [22, 24–26], but this may be ineffective because their bet-hedging life-history strategies mean that populations change relatively slowly over time [27]. Functional responses (intake rate in relation to food availability) may instead be more sensitive [28] but while traditional dietary analyses may reveal responses to landfill closures [25, 29], they often underrepresent soft prey items including anthropogenic foods [30]. In contrast, GPS tracking provides a much more sensitive monitoring tool because it recreates precise behaviours over relatively short time intervals [31]. For example, this makes it possible to accurately quantify foraging range and duration, which provides important information on foraging and environmental conditions [32]. Tracking can also help identify indirect ecological impacts arising from changes in gull space use [33, 34]. One possible outcome is that gulls may select new habitats, leading to potential conflict with humans [35, 36] or other wildlife [18, 37, 38]. Moreover, there is a well-defined analytical framework for analysing habitat selection [39, 40].

Here, we provide the first study of movement responses to landfill closure focussing on adult lesser black backed gulls (*Larus fuscus*) breeding at two colonies in northwest England. We study birds before and after the closure of two large open-air landfills (producing 150–200,000 tonnes of waste per annum). We test for changes in foraging behaviour and habitat selection to better understand any potential direct effects, as well as any possible indirect effects of habitat switching. In doing so, we not only increase our knowledge of the ecological consequences of landfill closures, but also how this may change the movement ecology of dependent animals.

## Methods

### Study sites and study period

Fieldwork was conducted during the breeding seasons (May-July) from 2014 to 2017 at two UK lesser black-backed gull colonies within Special Protection Areas (SPAs; EC Birds Directive 2009/147/EC) where breeding lesser black-backed gulls are a designation feature.

Ribble Marshes in Lancashire ( 53.7^o^, -2.98^o^; hereafter “Ribble”), is a stable mixed colony of herring (*Larus argentatus*) and lesser black-backed gulls (6554–7022 breeding pairs in 2016) in saltmarsh and rank vegetation. Anecdotal observations and visualisation of foraging tracks revealed that lesser black-backed gulls breeding at Ribble frequently visited Arpley Tip (53.4^o^, -2.62^o^), a large landfill with a solid waste capacity of c. 200,000 tonnes p.a., 43 km south-southeast of the colony that closed in December 2016 (http://myplanning.warrington.gov.uk).

South Walney nature reserve in Cumbria (54.7^o^, -3.23^o^; hereafter “Walney”) lies 41.5 km NNW of Ribble. Walney is a mixed breeding colony of herring, great black-backed (*Larus marinus*) and lesser black-backed gulls, the latter having declined from an estimated 19,487 apparently occupied nests (AONs) during 1998–2002 to 1,981 AONs in 2018 (https://app.bto.org/seabirds/public/data.jsp). As with Ribble, visualisation of foraging revealed that lesser black-backed gulls breeding at Walney made frequent foraging trips to another large landfill; Jameson Road Landfill Site (53.9^o^, 3.02^o^), with a capacity of c. tonnes p.a. lies 19.5 km south-southeast of the colony and 64.4 km north-northwest of Arpley Tip, and closed in April 2017 (http://planningregister.lancashire.gov.uk/).

### GPS-tagging

Breeding adult lesser black-backed gulls were caught at the nest using wire mesh walk-in cage traps and then fitted with a solar-powered Global Positioning System (GPS) tag (either University of Amsterdam Bird‐Tracking System (UvA-BiTS) device or Movetech Flyway-18 GPS-GSM device) which collected regular positional fixes ([41]; Table S1, Additional File 1). UvA tags had greater overall functionality, allowing them to collect data at higher resolution than Movetech tags (5 min resolution vs. 60 min), requiring re-sampling of the data to allow comparison.

Devices were attached using a Teflon wing-loop harness, to facilitate long-term deployment without impacting breeding success or survival [42, 43]. “Permanent” harnesses were replaced with those containing a “weak-link” from 2017 allowing tag detachment without recapture (Table S1, Additional File 1). Device and attachment combinations were below the 3 % body mass recommended at the time ([44]; Table S2, Additional File 1). Although recent work suggests such percentage of body mass thresholds may be inappropriate for mitigating device effects [45], previous work showed no negative influence of this type of tag attachment [42, 43]. This tagging work resulted in a sample of 48 individual adult lesser black-backed gulls which provided data in the years before and after landfill closure (Table 1). All tagging was performed under license from the British Trust for Ornithology’s independent Special Methods Technical Panel of the UK ringing scheme. All tagged individuals were fitted with uniquely engraved colour rings for subsequent field identification.

**Table 1:** Sample sizes of tagged adult lesser black-backed gulls at each colony before and after landfill closure, following subsampling and removal of incomplete trips. Some individual gulls were tracked in both years of the study (Ribble = 3, Walney = 18); these totals are shown in brackets in the year 2017 row for each site

Colony	Year	Landfill Status	Number of Individuals
Ribble	2016	Open	5
Ribble	2017	Closed	13 (3)
Walney	2016	Open	33
Walney	2017	Closed	18 (18)

### Data analysis

#### Foraging behaviour

We limited movement analysis to GPS data during 4-19th June (to coincide with late incubation/early chick-rearing) in both 2016 (immediately before closure) and 2017 (immediately after closure). Tracks were resampled to a one-hour resolution, due to differences in sampling frequency between tags. Foraging trips were defined as any positional fix outside the colony boundary (Figs S1 & S2, Additional File 1) with no data gaps greater than four hours (i.e. with good satellite coverage). Colony boundaries represent habitat available within the immediate vicinity of the nesting area for non-foraging activity, such as preening, loafing and bathing, based on field observations. Due to low data resolution, we were unable to distinguish between resting and foraging behaviours, therefore we assumed that all absences from the colony represent foraging trips.

For each foraging trip we calculated duration (hours), total length (straight-line point to point distance km) and distance to distal point (km). In order to test for an effect of landfill closures on gull foraging behaviour, we then modelled these response variables separately, using GLMMs with a gamma distribution and log link function. Due to convergence issues with the trip length models, we log-transformed this response variable and instead fit linear mixed effects models with a Gaussian error structure. For each response variable we created maximal models containing landfill status (open vs. closed), colony and the two-way interaction between colony and landfill status as fixed effects. To account for pseudoreplication resulting from repeat observations, we included individual identity as a random intercept.

#### Landfill utilisation

For each foraging trip, we classified all fixes into one of seven habitat categories (agriculture, coastal, freshwater, landfill, marine, other, urban; Table S3, Additional File 1) using the 100 m resolution Corine European Landcover raster database (available at: https://land.copernicus.eu/pan-european/corine-land-cover/clc2018) overlaid with active landfill sites (Environment Agency. Permitted Waste Sites - Authorised Landfill Site Boundaries - https://data.gov.uk).

To investigate whether there was a difference in the relative use of the focal landfills compared to other landfills in the years before and after closure, we first classified all landfill fixes as either “focal” (Arpley Tip or Jameson Road) or “other” and then calculated both the total number and proportion of fixes in “focal” and “other” landfills in each year.

We then quantified changes in overall landfill utilisation following landfill closure. The habitat at the distal point (a proxy for foraging habitat; [46]) of each foraging trip was classified as either “landfill” or “other”. To examine whether landfill closure influenced the probability of an individual gull foraging at any landfill site on a given foraging trip, we modelled landfill visits using a generalised linear mixed effects model (GLMM) with a binomial error structure. The full model contained site, landfill status and the two-way interaction as fixed effects and individual as a random intercept.

#### Habitat selection

We modelled resource selection functions (RSFs) that account for differences in habitat availability by comparing visited habitats with randomly generated pseudoabsences [39]. We first removed all birds with < 5 location fixes in a given year. For each fix, we generated five pseudo-absences within the 100 % minimum convex polygon (MCP) of the colony in that year. This allowed us to adequately capture the composition of available habitat within the foraging range, including rare habitats like landfills, without models becoming too computationally intensive [47].

Based on observed habitat use, we modelled RSFs for the five most visited foraging habitats (agriculture, coastal, landfill, marine and urban; Fig. S3, Additional File 1), fitting separate models for each site and year. For each foraging habitat, probability of gull utilisation was modelled as a function of habitat type (focal habitat vs. other), breeding site and the two-way interaction using binomial generalised linear models (GLM) with a logit link. Random intercepts are often fitted in linear models to deal with repeated individual measures [48]. However in this instance, we do not fit a random intercept for each individual, as this simply represents the ratio of real location points to pseudoabsences, which is constant [49]. In all models we assigned a weighting of 5 to real location points, proportional to the ratio of real locations to pseudoabsences in the data set [50]. A significant interaction effect supports the hypothesis that habitat selection varies with landfill status. Model fit was assessed by calculating AUC [51], predictive power, sensitivity and specificity (52; Table S4, Additional File 1).

#### Body condition

All captured birds were measured (wing length, bill depth, bill length and total head and bill length (mm)) and weighed (using a Pesola spring balance to the nearest 10 g). Morphometric data were used when considering tag effects (Table S2, Additional File 1) and to calculate adult body condition. For adults breeding at Ribble (2016 landfill open *n* = 19 and 2017 landfill closed = 21), we calculated the scaled mass index (Mi), which standardises body mass at a fixed value of a linear body length measurement (here, wing length, the structural measurement most correlated with mass) [53]. To test for an effect of landfill closure, we compared mean adult body condition in the years before and after closure using a Welch’s two-sample t-test for unequal variances. This comparison was made between two separate groups of birds, with no individuals measured in both years.

#### Model selection

Model selection was based on AIC, with the model with the lowest AIC value selected for all analyses (Tables S5 – S8, Additional File 1). Normality plots and visualisation of residuals were used to check assumptions for normality and homogeneity of variance. For mixed effects models, we calculated both marginal (MR^2^) and conditional (CR^2^) r-squared values [54], using the trigamma function where available [55]. All statistical analyses were conducted in R (v3.6.2, [56]).

## Results

### Foraging behaviour

**Fig. 1:**
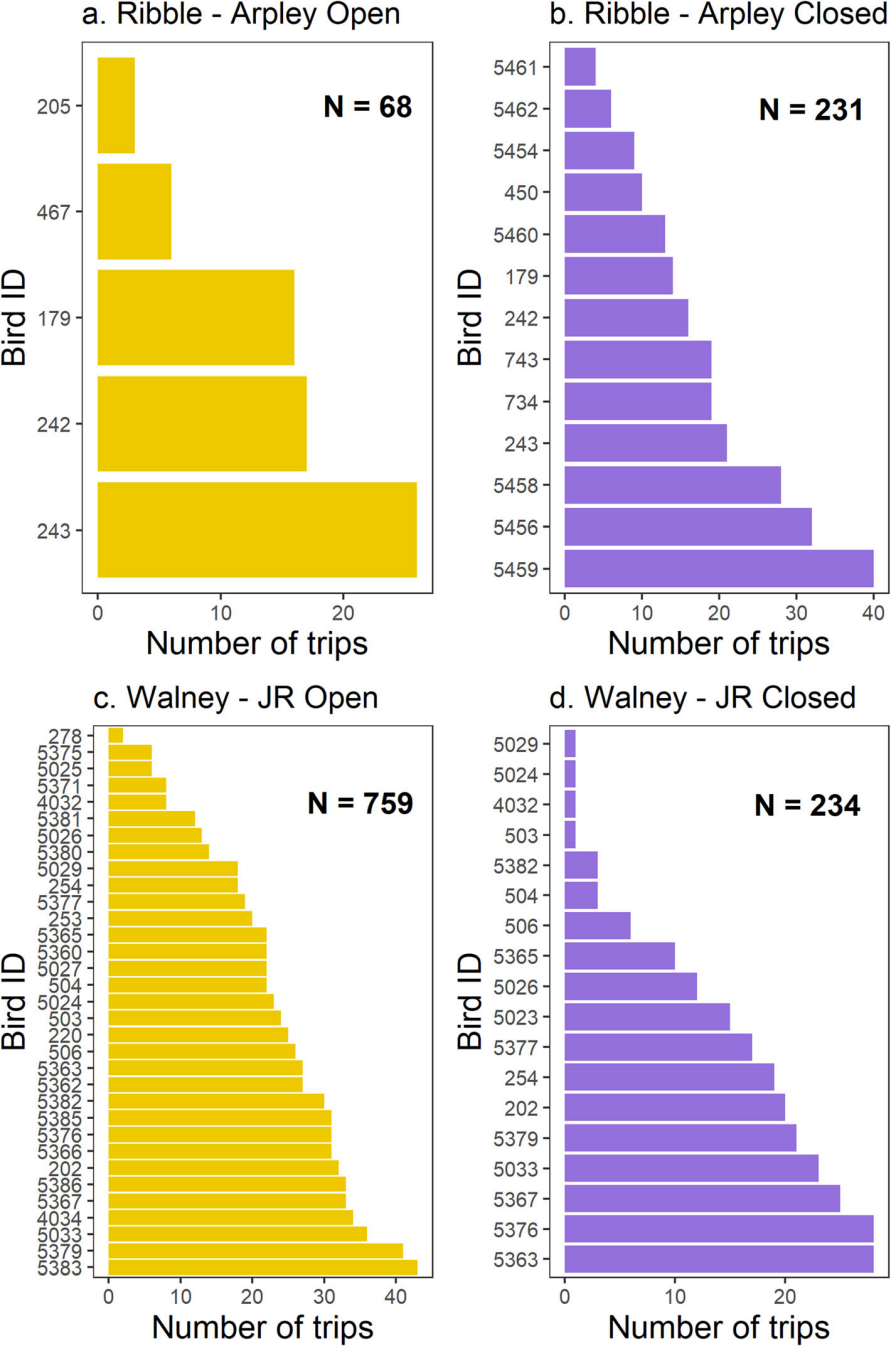
Number of complete foraging trips for each individual GPS-tracked lesser black-backed gull breeding at Ribble and Walney in the years before and after closure of the focal landfills (Ribble = Arpley Tip; Walney = Jameson Road Landfill). Number of individual foraging trips ranged between a minimum of 1 and a maximum of 43. N denotes the total number of complete foraging trips recorded at each colony in each year

We recorded 1,292 complete foraging trips from 48 individual GPS-tagged lesser black-backed gulls. The annual number of foraging trips per individual varied between 1 and 43 (Fig. 1).

**Fig. 2:**
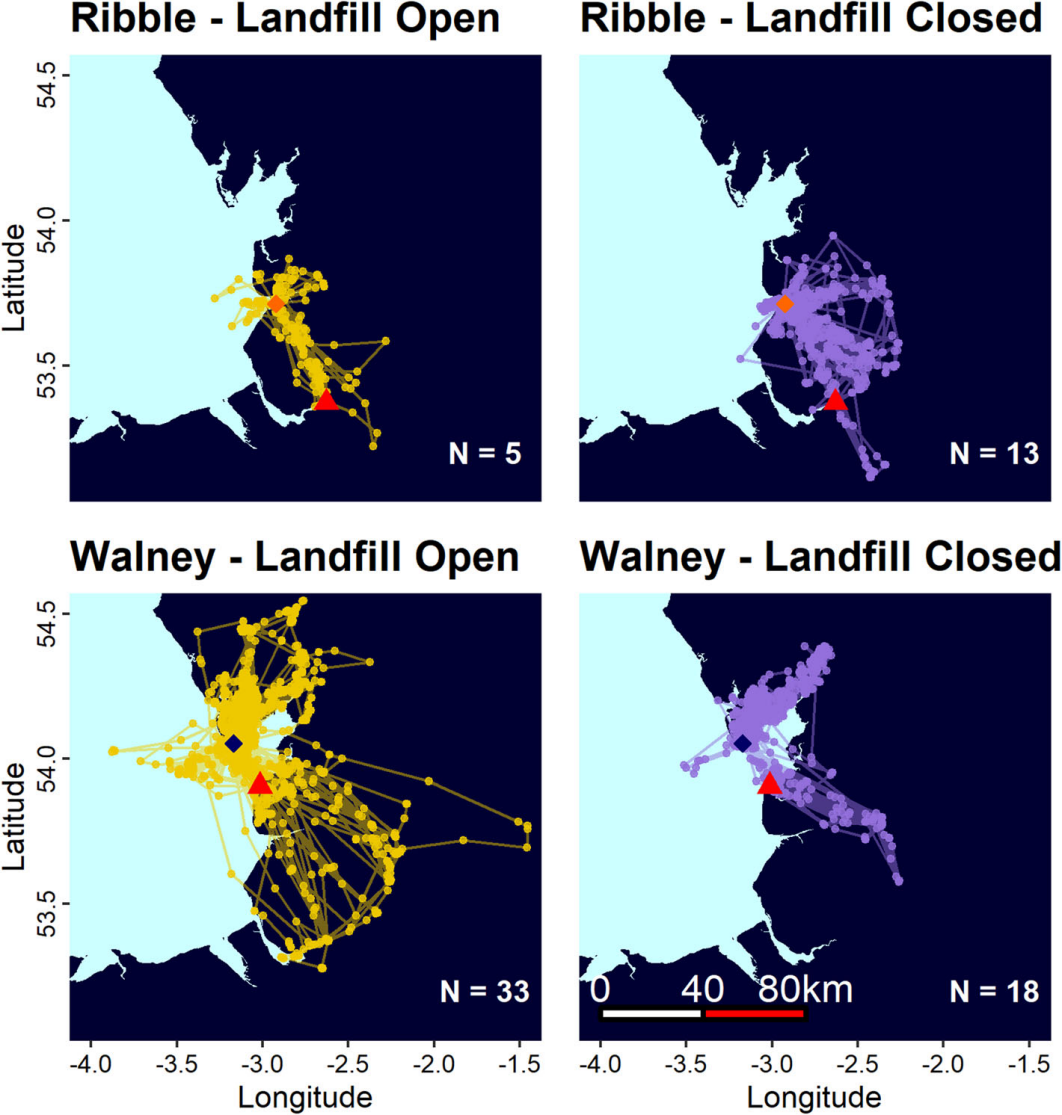
Complete foraging trips by all tracked lesser black-backed gulls from Walney and Ribble before (yellow) and after (purple) landfill closures. N denotes the number of birds tracked in each year (For number of foraging trips see Fig. 1). Colony locations are marked with navy (Walney) or orange (Ribble) diamonds. Focal landfills, Jameson Road Landfill (Walney) and Arpley Tip (Ribble) are marked with red triangles

Generally, breeding adult lesser black-backed gulls (*n* = 48) from both sites travelled inland to forage, either to the southeast or northeast of the colony and largely avoided marine habitats and there was some overlap between colonies (Fig. 2).

**Fig. 3:**
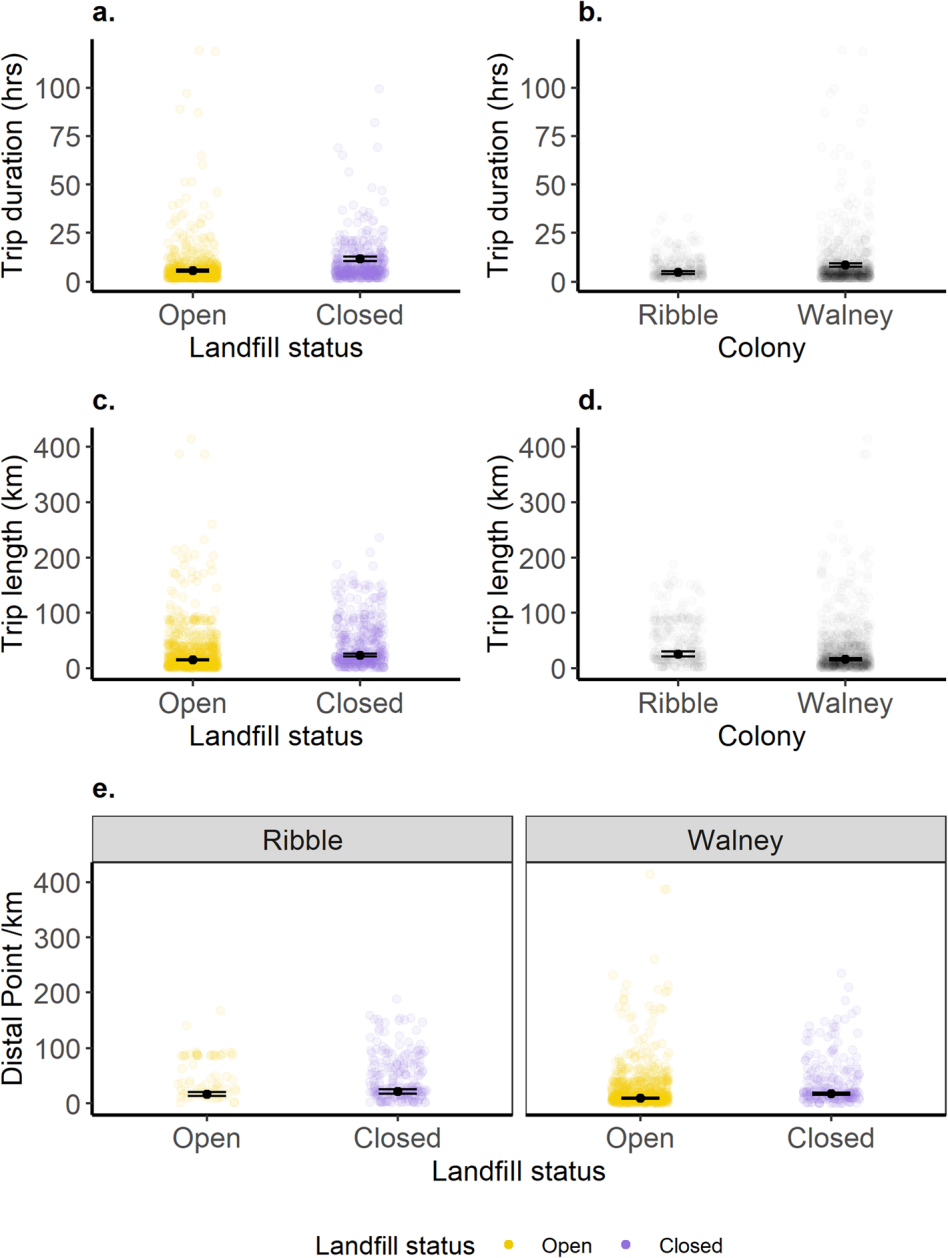
Back-transformed model estimates and SEs from the best supported models explaining variation in foraging trip characteristics from breeding lesser black-backed gulls at the colony-level. **a**) Trip durations (hrs) were greater following the closure of the focal landfill across both sites. **b**) Trip durations were longer for birds at Walney than those breeding at Ribble regardless of landfill status. **c**) Trip lengths (km) were greater when the landfill was closed than when it was open across both sites. **d**) Trip lengths were greater at Ribble than Walney. **e**) Distal points (km) were further following the closure of the focal landfill, with a greater increase in distal point following landfill closure at Walney compared to Ribble

Breeding gulls increased the distance travelled and duration of foraging trips at both sites following landfill closures. Estimated mean foraging trip durations increased from 5.7 h (95 % CIs: 4.7–6.8 h) when the landfill was open to 11.7 h (95 % CIs: 9.7–14.2 h) when the landfill was closed (Fig. 3a; Table S9, Additional File 1). Similarly, estimated mean trip length increased from 15.0 km (95 % CIs: 12.3–18.2 km) to 23.5km (95 % CIs: 18.8–29.4 km) following landfill closure (Fig. 3c; Table S9, Additional File 1). Estimated mean distal point distance increased from 9.7 km (95 % CIs: 7.8–12.1 km) to 17.4 km (95 % CIs: 13.6–22.3 km) at Walney, and from 16.4 km (95 % CIs: 10.9–24.5 km) to 21.2 km (95 % CIs: 15.1–29.7 km) at Ribble (Fig. 3e). This latter shift was stronger at Walney than Ribble due to the interaction between landfill status and colony (Table S9, Additional File 1).

We also observed differences in foraging behaviour between colonies, with Walney breeders having longer trip durations, but travelling shorter distances than birds from Ribble (Fig. 3; Table S9, Additional File 1).

### Landfill utilisation

**Fig. 4:**
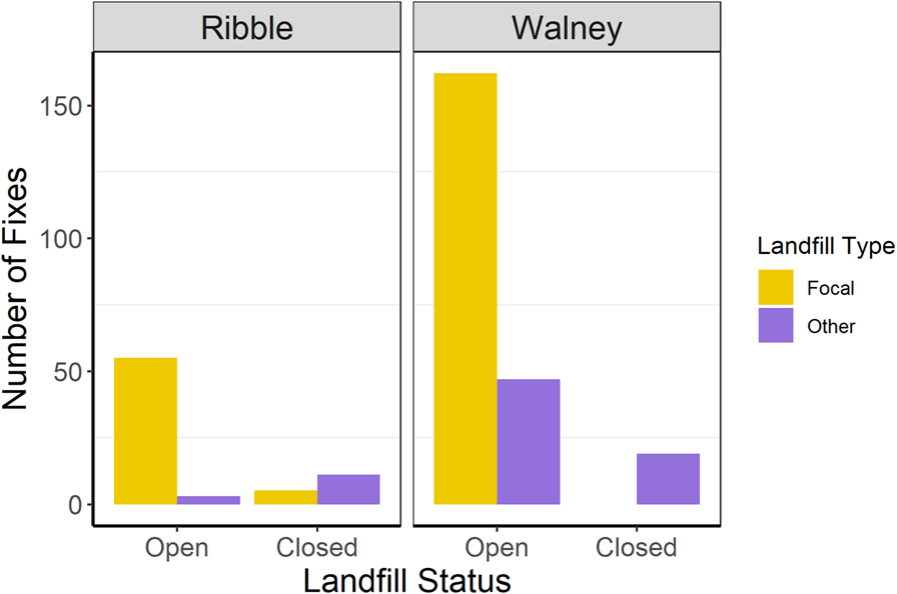
Total number of GPS fixes from tagged lesser black-backed gulls within focal (yellow) and other (purple) landfills in the years before and after landfill closure. At both colonies, the overall number of landfill fixes dropped dramatically following closure of the focal site. Birds from Ribble occasionally visited Arpley Tip following closure whilst birds from Walney ceased visiting Jameson Road Landfill Site altogether

**Fig. 5:**
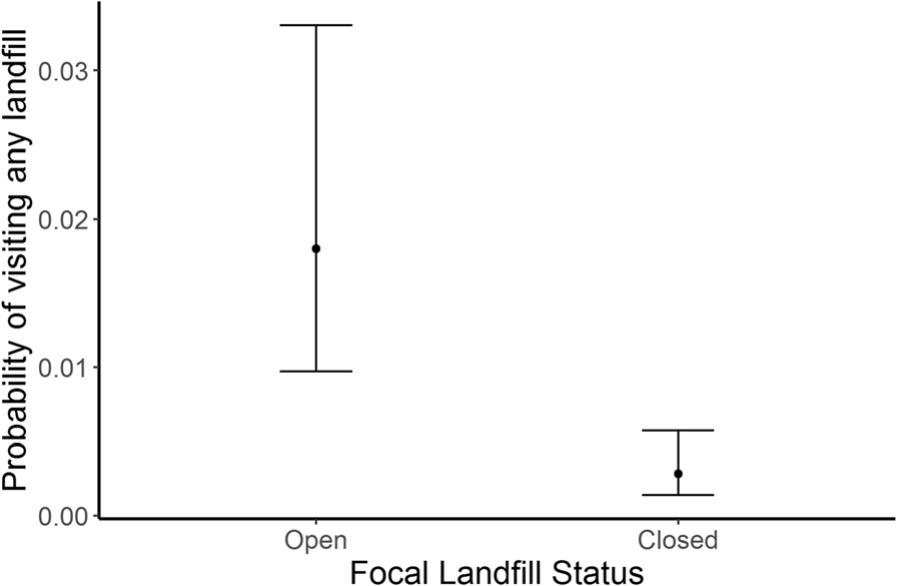
Probability of foraging at any landfill site on a given foraging trip for individual breeding lesser black-backed gulls in the years before and after closure of the focal landfills. Birds breeding at Ribble and Walney were more likely to visit landfills in the year before the closure of the focal landfill (Arpley and Jameson Road respectively)

When operational in 2016, the focal landfills fixes were by far the most frequently visited sites at both Ribble (94.8 % of landfill fixes) and Walney (77.5 % of landfill fixes). However, following landfill closure in 2017 birds from Walney ceased to visit Jameson Road, although Ribble birds occasionally visited Arpley (31.3 % of landfill fixes) even after its closure. At both sites, the total landfill fixes were dramatically reduced following the landfill closure (Fig. 4). At the colony-level, individual birds were less likely to forage at any landfill site on a given foraging trip following the closure of the focal landfill (mean difference ± SE = -1.87 ± 0.43; Fig. 5).

### Habitat selection

**Fig. 6:**
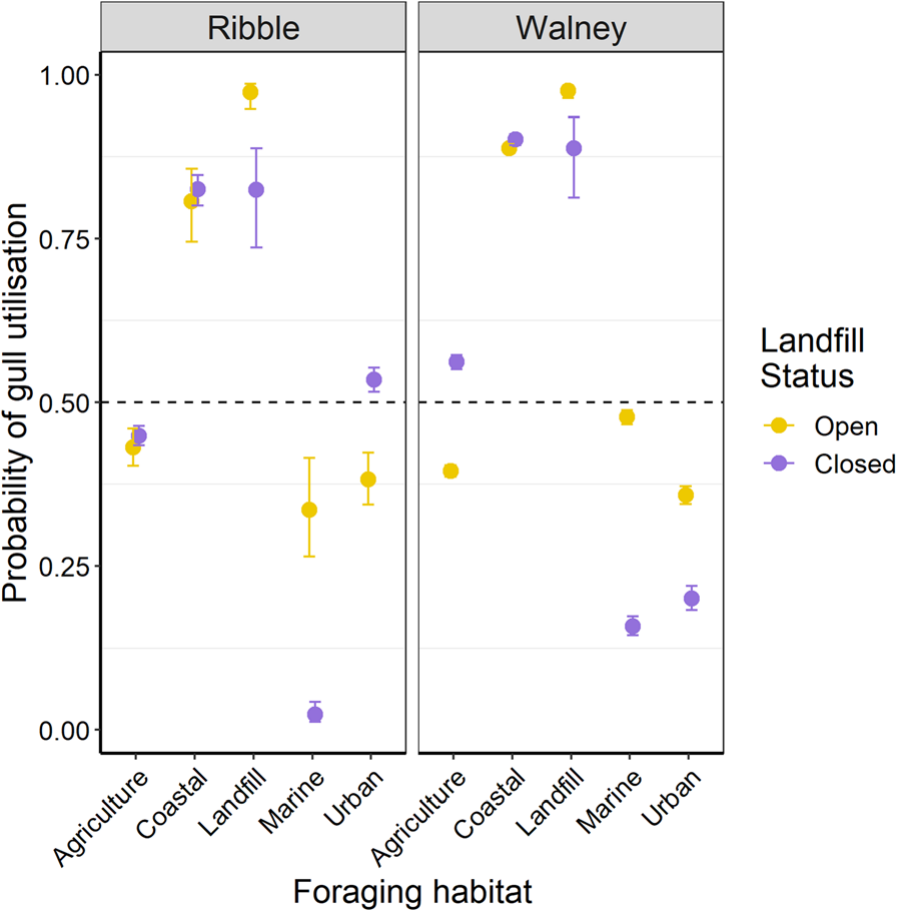
Estimates and 95 % confidence intervals from resource selection models for all GPS-tracked lesser black-backed gulls breeding at Ribble and Walney before (yellow) and after (purple) focal landfill closure. Models estimate the probability that a given location point represents a real GPS location rather than a pseudo-absence in response to five main foraging habitat categories (agriculture, coastal, landfill, marine, urban) – i.e. habitat selection - the probability that gulls are using that habitat relative to its availability. A probability of 0.50 indicates that birds used habitat in proportion to its availability whilst values of > 0.50 indicate selection for that habitat type at the colony-level

The probability of an individual gull foraging at any landfill on a given trip declined at both colonies following closure of the focal site, however this probability was much more variable in the year prior to closure, suggesting landfills were not visited by all individuals or on all foraging trips (Fig. 5). Although selections for landfills declined overall, they remained an important foraging destination and were still actively selected for relative to their availability. Ribble breeders increased their utilisation of urban habitats following landfill closure whilst birds from Walney used urban habitats less frequently, instead increasing their selection for agricultural habitats. At both colonies, selection for marine habitats was weaker following the landfill closure, however selection for coastal habitats remained high at both colonies across both years of the study (Table 2; Fig. 6).

**Table 2:** Estimates for the effect of an interaction between the habitat variable and breeding site on the probability of a location being a real gull location or a pseudo-absence. Delta (Δ) AIC refers to the change in AIC caused by removing the interaction. Δ AIC values > 2, suggest that selection for that habitat type differs significantly following landfill closure. Stars next to *p*-values represent significance levels (* < 0.05; ** < 0.01; *** < 0.001)

Habitat Variable	Site	Estimate for landfill status interaction	*p* value	Δ AIC
Agriculture	Ribble	0.14 (± 0.09)	0.143	0.2
Coastal	Ribble	0.18 (± 0.21)	0.380	-1.2
Landfill	Ribble	-2.29 (± 0.45)	< 0.001^***^	26.1
Marine	Ribble	-3.11 (± 0.37)	< 0.001^***^	99.8
Urban	Ribble	0.83 (± 0.11)	< 0.001^***^	58.2
Agriculture	Walney	1.02 (± 0.04)	< 0.001^***^	752.4
Coastal	Walney	0.24 (± 0.06)	< 0.001^***^	12.1
Landfill	Walney	-1.68 (± 0.37)	< 0.001^***^	14.8
Marine	Walney	-1.76 (± 0.06)	< 0.001^***^	942.6
Urban	Walney	-0.85 (± 0.07)	< 0.001^***^	162.9

### Adult body condition

There was no significant effect of landfill status on adult body condition at Ribble (Mi) (t = -0.56, df = 32.548, *p* = 0.577). A power analysis, using the difference between group means (0.56) as the effect size, revealed a relatively low power of 41.1 %.

## Discussion

Our aim was to quantify the impact of landfill closures on gull movement ecology. We found that gulls tracked from two different colonies travelled further, both in terms of total trip length (13.5 km) and maximum distance from the colony (Ribble = 4.8 km; Walney = 7.7 km) and spent longer (6.1 h) on foraging trips following landfill closures (Fig. 3). Moreover, while some birds were able to find alternative landfills (Fig. 5), they increased use of agricultural habitats or urban areas, depending on colony (Table 2; Fig. 6). We discuss the implications of waste management on animal movement and in turn their conservation implications below.

### Foraging Behaviour

Landfills provide predictable food [3], although this is clearly not the case if they close. Trip duration increased at both colonies over the course of this study (Fig. 3a) suggesting birds spent more time travelling, searching for and/or handling food. While we cannot completely exclude other environmental impacts [57], it seems most likely that the observed changes were due to the landfill closures. Birds may be searching for other landfills or since they use landfills for navigation [15], closures could impact searching. Habitat switching also likely played a role (Fig. 6). Agricultural habitats provide low quality foods [58, 59], while urban areas may have more ephemeral [34] and less predictable foraging opportunities [14, 36].

Alternatively, longer foraging trips may be a direct consequence of breeding failure, which removes the constraint to act as central place foragers [60]. However, this seems unlikely to explain our results because, while observational visits revealed there were high levels of reproductive failure at the colony-level at Ribble in 2017 (due to flooding), breeding success appeared to be similar between years at Walney. Whatever the reason, longer foraging trips mean longer absences from the nest site, which could increase the risk of chick mortality or conspecific incursions into breeding territories [15].

### Habitat selection

While gulls were able to find alternative landfills following closure of two large landfill sites they still showed a sharp decline in overall landfill use and instead, selected either agricultural (Walney) or urban (Ribble) habitats (Fig. 6). This suggests gulls were able to rapidly alter their behaviour in response to changing resource availability and locate new foraging sites, which mirrors observations of behavioural flexibility in other gull populations [29, 61]. Such flexibility is not universal among gulls however, with several studies demonstrating a high degree of individual foraging specialisation [59, 62, 63]. Moreover GPS-tracking of lesser black-backed gulls which underwent forced colony relocation showed that birds did not optimally adapt to the food landscape at the new colony, leading to increased foraging effort and negative consequences for offspring development [64]. Our findings also highlight the value of fine-scale tracking for monitoring short-term effects of changing waste management, that demographic and dietary studies may overlook.

The selection of alternative foraging habitats could relate to colony-level differences in habitat availability, with more available urban habitats at Ribble (29 % pseudoabsences) than Walney (15 % pseudoabsences). Moreover, Walney birds did not switch to forage in the nearby town of Barrow-in-Furness, possibly due to competitive exclusion by an already present urban breeding population [65, 66]. Longer journey times may therefore make urban foraging unprofitable for Walney birds, prompting a shift to agricultural habitats.

Another striking result from this analysis was the reduction in marine foraging following landfill closure (Fig. 6). Lesser black-backed gulls often forage extensively offshore when breeding [67, 68], although our study gulls spent little time at-sea. This may be due to high value urban foraging opportunities in the vicinity of the colony at Ribble [69], or possibly a degraded local marine environment around both colonies, with for example, fewer fishery discards [70]. Alternatively, high rainfall in 2017 could have made agricultural soil invertebrates more accessible [57].

### Methodological considerations

We included all foraging trip points in our analysis (1 h fixes) which risks including commuting and not just foraging locations. To address this potential issue, we repeated the analysis with just distal trip locations, which is most likely where central place foragers feed [71] but found similar results (Tables S10 & S11; Fig S4, Additional File 1).

One potential shortcoming is the relatively low predictive power of our habitat selection models (Table S4, Additional File 1). AUC values were below the optimal 0.8 [52, 72],suggesting unexplained variation possibly due to intrinsic factors such as sex [73], reproductive status [68, 74], and individual preferences [15, 59, 62], and extrinsic factors such as weather [57] and food availability [75]. Nevertheless, AUC values were greater than 0.5 indicating at least, better than random support for an effect of landfill closures, even without controlling for a range of potential confounding effects.

### Body condition

We found no closure effects on adult body condition at Ribble. This contrasts with work on yellow-legged gull (*Larus michahellis*) showing a negative impact of landfill closures on body condition [26], although our low power (41.1 %) suggests the likelihood of a Type II error. Alternatively, adult gulls may transfer the costs of landfill closures to their offspring, in favour of self-maintenance as has been found in other studies of landfill closures and discards bans [22, 24, 76, 77].

Another possibility is that switching to alternative habitats and/or the fact that not all individuals foraged at focal dumps, means gulls can buffer any effects of the landfill closure. Finally, adult body condition may be influenced by other factors such as carry-over effects [78].

### Animal tracking and anthropogenic change

The movement responses to landfill closures were only detectable via continuous gull tracking, highlighting the value of remotely downloadable precision movement data for applied research [79]. GPS-loggers used here provided hourly (or higher) resolution [73, 81, 83], in contrast to the much longer temporal windows of diet and demographic studies. Moreover, long-term deployments allow the detection of non-linear responses to anthropogenic perturbations [82].

Despite these advantages there are trade-offs. Bio-logging devices may be costly relative to dietary or demographic monitoring, resulting in smaller sample sizes, although they are becoming cheaper. Moreover, designation of conservation status and subsequent interventions are generally initiated by population change [83]. The lability of gull foraging behaviour may buffer fitness consequences of landfill closures [61, 73] and further work is required to link changes in movement ecology to demographic change.

### Conservation and management implications

Here we demonstrate how gulls breeding in two colonies designated as SPAs forage at landfills outside protected area boundaries, highlighting the importance of spatial scale for mobile species in management decisions [84, 85]. Despite their importance, we found no effect of landfill closure on body condition, possibly indicating resilience to anthropogenic perturbations [61], although we cannot rule-out other impacts [15, 64]. Future work should combine GPS-tracking with individual monitoring to better understand any potential fitness consequences [64].

Although demographic responses to changing PAFS may be slow [28], our movement data demonstrate rapid behavioural responses [25, 29]. This could alter the intensity and spatial-distribution of human-gull conflict, necessitating conservation or management interventions [70, 86]. For example, western gulls (*Larus occidentalis*), in California subsidised by landfills, have imperilled local salmon populations [18, 87].

In our study, increased agricultural foraging may create conflict with farmers via consumption of crop seeds or livestock feed and the risk of disease transmission to livestock [7, 35, 59]. Alternatively, more frequent urban foraging could increase nuisance behaviour [36, 88]. However landfill closures may also provide management benefits, reducing the need for deterrents and lethal management required to prevent gulls from foraging at active dumps [89, 90].

## Conclusions

GPS tracking revealed how gull movement ecology is shaped by the availability of landfill as foraging habitat. The long-term consequences of closures are unclear but in the short-term gulls respond by rapidly shifting to new foraging areas. This may shift human-gull conflicts to urban and agricultural habitats. We conclude that tracking animal movement can and should be used to understand ecological consequences of anthropogenic change. Future work should combine these approaches with demographic monitoring to quantify the consequences of changing gull movement behaviour in terms of fitness and demographic change.

## Supplementary Information

**Additional file 1: Table S1.** A summary of number of tags deployed, device type and attachment method in each year at both colonies. **Table S2.** Tag and harness mass as a percentage of body mass for all birds tagged at both colonies between 2014 and 2018. Tag and harness mass includes 3g for a colour-ring. For birds some birds tagged with Movetech devices in 2018 (*) the exact tag masses are missing and therefore values represent closest assumed tag masses of 24.5g based on known masses of tags from that year. This value of total tag and harness mass includes 3g for colour ring and 3.5 for harness. **Figure S1.** Colony area used to define foraging trips for Ribble birds (red polygon). Any GPS fixes outside the colony boundary were defined as a foraging trip. **Figure S2.** Colony area used to define foraging trips for Walney birds (blue polygon). Any GPS fixes outside the colony boundary were defined as a foraging trip. **Table S3.** Habitat classifications from the Corine European Landcover database grouped into main foraging habitat types used for habitat selection modelling.abita. **Figure S3.** Proportion of real gull location fixes vs. randomised pseudoabsences (Pseudo) assigned to each of seven main habitat classes – agricultural, coastal, freshwater, landfill, marine, urban and other (scrub woodland and other non-foraging habitats) – for gulls breeding at Ribble and Walney in the years before and after landfill closure. Available habitat at Ribble was dominated by agricultural and urban environments whilst marine and freshwater habitats constituted a greater proportion of the pseudoabsence locations for Walney. **Table S4.** Assessment of habitat selection models containing a habitat variable, site and the site*habitat interaction for all tagged birds (Table 2). All metrics are derived from a confusion matrix based on the original data. AUC (area under the receiver operating curve) ranges from 0 to 1, where 0.5 is random, and higher values indicate better model performance. CC = Correct Classification, PPP = Positive Predictive Power, NPP = Negative Predictive Power, Sen. = Sensitivity, Spec, = Specificity. **Table S5.** Top five candidate models to explain probability of visiting any landfill at the colony-level ranked by AIC weight. Pseudo-R^2^ values for the selected model - MR^2^= 0.0399, CR^2^ = 0.328. **Table S6.** Top five candidate models to explain trip duration (hrs) at the colony-level ranked by AIC weight. Pseudo-R^2^ values for the selected model - MR^2^ = 0.0833, CR^2^ = 0.299. **Table S7.** Top five candidate models to explain trip length (km) at the colony-level ranked by AIC weight. Pseudo-R^2^ values for the selected model - MR^2^ = 0.067 CR^2^= 0.271. **Table S8.** Top five candidate models to explain distal point (km) at the colony-level ranked by AIC weight. Pseudo-R^2^ values for the selected model - MR^2^ = 0.0764, CR^2^ = 0.269. **Table S9.** (Generalised) Linear Mixed Model estimates ± standard error for foraging trip duration (hrs), trip length (km) and distal point distance (km) for lesser black-backed gulls in relation to landfill status and colony, with bird ID fitted as a random intercept, Estimates are from the model with the lowest AIC (see Tables S6 – S8). **S10.** Estimates for the effect of an interaction between the habitat variable and landfill status on the probability of a location being a real gull location or a pseudo-absence based on distal foraging trip locations only. Delta (Δ) AIC refers to the change in AIC caused by removing the interaction. If Δ AIC > 2, the interaction is not significant meaning we found no evidence for an effect of breeding habitat on selection for that habitat type. If the interaction effect is significant, habitat selection varied with landfill status. Models were run separately for each site. Stars next to *p*-values represent significance levels (* < 0.05; ** < 0.01; *** < 0.001). **Figure S4.** Estimates and 95% confidence intervals from resource selection models for all GPS-tagged lesser black-backed gulls breeding at Ribble and Walney before (gold) and after (purple) closure of the focal landfill site based on distal trip locations. Models estimate the probability of a given location point being a real gull location rather than a pseudo-absence in response to five main foraging habitat categories (agriculture, coastal, landfill, marine, urban). A probability of 0.50 indicates that birds used habitat in proportion to its availability whilst values of > 0.50 indicate selection for that habitat type at the colony-level. **Table S11.** Assessment of habitat selection models for distal foraging trip locations containing a habitat variable, site and the site*habitat interaction (Table S15). All metrics are derived from a confusion matrix based on the original data. AUC (area under the receiver operating curve) ranges from 0 to 1, where 0.5 is random, and higher values indicate better model performance. CC = Correct Classification, PPP = Positive Predictive Power, NPP = Negative Predictive Power, Sen. = Sensitivity, Spec, = Specificity.

## Data Availability

The UvA-BiTS tracking studies are facilitated by infrastructures for e‐Science, developed with support of the NLeSC (http://www.esciencecenter.com/) and LifeWatch, carried out on the Dutch national e‐infrastructure with support from the SURF Foundation. The data are held jointly by the BTO, University of Amsterdam and the funders of the project, and can be made available through their agreement. The datasets collected using Movetech tags during this study are stored at Movebank (www.movebank.org) within the following repositories: “BTO - Barrow 2017 - Lesser Black-backed Gull” (ID, 277,843,654), “BTO - Barrow 2018 - Lesser Black-backed Gull” (ID, 482,136,669), “BTO North West England 2016 - Lesser Black-backed Gull” (ID, 167,983,392), “BTO Ribble Estuary 2017 - Lesser Black-backed Gull” (ID, 277,841,852), “BTO Ribble Estuary 2018 - Lesser Black-backed Gull” (ID, 482,136,485), and “BTO Ribble Estuary 2019 - Lesser Black-backed Gull” (ID, 849,740,134). The data are held jointly by the BTO and the funders of the project and can be made available through agreement upon request. Biometric data are held in the UK National Ringing Scheme database and are available from the BTO on request.
