# Supplementary material for: GPS tracking reveals landfill closures induce higher foraging effort and habitat switching in gulls
Source: Mov Ecol. 2021 Nov 12;9:56. doi: 10.1186/s40462-021-00278-2 (PMC8588598; doi:10.1186/s40462-021-00278-2)
Supplement: Supplementary file 1 — Additional file 1: Table S1. A summary of number of tags deployed, device type and attachment method in each year at both colonies. Table S2. Tag and harness mass as a percentage of body mass for all birds tagged at both colonies between 2014 and 2018. Tag and harness mass includes 3g for a colour-ring. For birds some birds tagged with Movetech devices in 2018 (*) the exact tag masses are missing and therefore values represent closest assumed tag masses of 24.5g based on known masses of tags from that year. This value of total tag and harness mass includes 3g for colour ring and 3.5 for harness. Figure S1. Colony area used to define foraging trips for Ribble birds (red polygon). Any GPS fixes outside the colony boundary were defined as a foraging trip. Figure S2. Colony area used to define foraging trips for Walney birds (blue polygon). Any GPS fixes outside the colony boundary were defined as a foraging trip. Table S3. Habitat classifications from the Corine European Landcover database grouped into main foraging habitat types used for habitat selection modelling.abita. Figure S3. Proportion of real gull location fixes vs. randomised pseudoabsences (Pseudo) assigned to each of seven main habitat classes – agricultural, coastal, freshwater, landfill, marine, urban and other (scrub woodland and other non-foraging habitats) – for gulls breeding at Ribble and Walney in the years before and after landfill closure. Available habitat at Ribble was dominated by agricultural and urban environments whilst marine and freshwater habitats constituted a greater proportion of the pseudoabsence locations for Walney. Table S4. Assessment of habitat selection models containing a habitat variable, site and the site*habitat interaction for all tagged birds (Table 2). All metrics are derived from a confusion matrix based on the original data. AUC (area under the receiver operating curve) ranges from 0 to 1, where 0.5 is random, and higher values indicate better model performance. CC = C [file 40462_2021_278_MOESM1_ESM.docx]

**Supplementary Information**

**Additional tagging information**

Captured birds were fitted with a solar‐powered GPS tag (either a University of Amsterdam Bird‐Tracking System (UvA-BiTS) device or a Movetech Flyway-18 GPS-GSM device) attached via a Teflon wing-loop harness. “Permanent” harnesses were replaced by a “weak-link” design from 2017 allowing the tag to detach without recapture (Table S1).

**Table S1.** A summary of number of tags deployed, device type and attachment method in each year at both colonies.

| **Site** | **Year** | **Birds Tagged** | **Tag type** | **Attachment** |
| --- | --- | --- | --- | --- |
| Ribble | 2016 | 11 | Movetech | Permanent harness |
| Ribble | 2017 | 2 | Movetech | Weak-link harness |
| Ribble | 2017 | 8 | UvA-BiTS | Weak-link harness |
| Walney | 2014 | 24 | UvA BiTS | Permanent harness |
| Walney | 2016 | 20 | UvA BiTS | Permanent harness |
| Walney | 2016 | 5 | Movetech | Permanent harness |

During tagging potential device effects were carefully considered. Although the accepted body mass threshold of 3% (36) is now debated, all tags fitted on gulls were below this threshold (Table S2).

**Table S2.** Tag and harness mass as a percentage of body mass for all birds tagged at both colonies between 2014 and 2018. Tag and harness mass includes 3g for a colour-ring. For birds some birds tagged with Movetech devices in 2018 (*) the exact tag masses are missing and therefore values represent closest assumed tag masses of 24.5g based on known masses of tags from that year. This value of total tag and harness mass includes 3g for colour ring and 3.5 for harness.

| **Bird ID** | **Tag Type** | **Site** | **Year** | **Tag and harness mass (g)** | **Bird weight (g)** | **Percentage of body mass (%)** |
| --- | --- | --- | --- | --- | --- | --- |
| 179 | Movetech | Ribble | 2016 | 25.25 | 920 | 2.74 |
| 205 | Movetech | Ribble | 2016 | 24.92 | 920 | 2.71 |
| 242 | Movetech | Ribble | 2016 | 25 | 920 | 2.72 |
| 243 | Movetech | Ribble | 2016 | 25.86 | 1010 | 2.56 |
| 446 | Movetech | Ribble | 2016 | 21.03 | 790 | 2.66 |
| 450 | Movetech | Ribble | 2016 | 21.43 | 800 | 2.68 |
| 464 | Movetech | Ribble | 2016 | 21.42 | 820 | 2.61 |
| 465 | Movetech | Ribble | 2016 | 21.01 | 780 | 2.69 |
| 467 | Movetech | Ribble | 2016 | 21.46 | 840 | 2.55 |
| 469 | Movetech | Ribble | 2016 | 21 | 890 | 2.36 |
| 484 | Movetech | Ribble | 2016 | 21 | 860 | 2.44 |
| 734 | Movetech | Ribble | 2017 | 19.26 | 980 | 1.97 |
| 743 | Movetech | Ribble | 2017 | 19.8 | 920 | 2.15 |
| 5454 | UvA S | Ribble | 2017 | 13.53 | 920 | 1.47 |
| 5456 | UvA S | Ribble | 2017 | 13.42 | 910 | 1.47 |
| 5458 | UvA S | Ribble | 2017 | 13.51 | 860 | 1.57 |
| 5459 | UvA S | Ribble | 2017 | 13.5 | 740 | 1.82 |
| 5460 | UvA S | Ribble | 2017 | 13.5 | 960 | 1.41 |
| 5461 | UvA S | Ribble | 2017 | 13.45 | 750 | 1.79 |
| 5462 | UvA S | Ribble | 2017 | 13.55 | 800 | 1.69 |
| 5463 | UvA S | Ribble | 2017 | 13.68 | 765 | 1.79 |
| 852 | Movetech | Ribble | 2018 | 17.8 | 880 | 2.02 |
| 853 | Movetech | Ribble | 2018 | 18.28 | 865 | 2.11 |
| 854 | Movetech | Ribble | 2018 | 17.96 | 845 | 2.13 |
| 862 | Movetech | Ribble | 2018 | 17.96 | 960 | 1.87 |
| 879 | Movetech | Ribble | 2018 | 18.1 | 970 | 1.87 |
| 880 | Movetech | Ribble | 2018 | 17.69 | 820 | 2.16 |
| 881 | Movetech | Ribble | 2018 | 17.76 | 920 | 1.93 |
| 882 | Movetech | Ribble | 2018 | 18.25 | 840 | 2.17 |
| 911* | Movetech | Ribble | 2018 | 24.5 | 1030 | 2.37 |
| 915* | Movetech | Ribble | 2018 | 24.5 | 955 | 2.56 |
| 494 | UvA L | Walney | 2014 | 18.50 | 870 | 2.13 |
| 496 | UvA L | Walney | 2014 | 18.50 | 720 | 2.57 |
| 497 | UvA L | Walney | 2014 | 18.50 | 780 | 2.37 |
| 499 | UvA L | Walney | 2014 | 18.50 | 860 | 2.15 |
| 501 | UvA L | Walney | 2014 | 18.50 | 990 | 1.87 |
| 502 | UvA L | Walney | 2014 | 18.50 | 710 | 2.61 |
| 503 | UvA L | Walney | 2014 | 18.50 | 660 | 2.80 |
| 504 | UvA L | Walney | 2014 | 18.50 | 730 | 2.53 |
| 506 | UvA L | Walney | 2014 | 18.50 | 720 | 2.57 |
| 4031 | UvA M | Walney | 2014 | 15.50 | 920 | 1.68 |
| 4032 | UvA M | Walney | 2014 | 15.50 | 710 | 2.18 |
| 4033 | UvA M | Walney | 2014 | 15.50 | 820 | 1.89 |
| 4034 | UvA M | Walney | 2014 | 15.50 | 940 | 1.65 |
| 4035 | UvA M | Walney | 2014 | 15.50 | 770 | 2.01 |
| 5023 | UvA S | Walney | 2014 | 13.50 | 680 | 1.99 |
| 5024 | UvA S | Walney | 2014 | 13.50 | 910 | 1.48 |
| 5025 | UvA S | Walney | 2014 | 13.50 | 930 | 1.45 |
| 5026 | UvA S | Walney | 2014 | 13.50 | 880 | 1.53 |
| 5027 | UvA S | Walney | 2014 | 13.50 | 760 | 1.78 |
| 5029 | UvA S | Walney | 2014 | 13.50 | 730 | 1.85 |
| 5030 | UvA S | Walney | 2014 | 13.50 | 900 | 1.50 |
| 5032 | UvA S | Walney | 2014 | 13.50 | 750 | 1.80 |
| 5033 | UvA S | Walney | 2014 | 13.50 | 790 | 1.71 |
| 5034 | UvA S | Walney | 2014 | 13.50 | 970 | 1.39 |
| 202 | Movetech | Walney | 2016 | 25.00 | 980 | 2.55 |
| 220 | Movetech | Walney | 2016 | 25.00 | 1100 | 2.27 |
| 253 | Movetech | Walney | 2016 | 25.00 | 980 | 2.55 |
| 254 | Movetech | Walney | 2016 | 25.00 | 900 | 2.78 |
| 278 | Movetech | Walney | 2016 | 25.00 | 960 | 2.60 |
| 5358 | UvA S | Walney | 2016 | 13.50 | 950 | 1.42 |
| 5360 | UvA S | Walney | 2016 | 13.50 | 790 | 1.71 |
| 5362 | UvA S | Walney | 2016 | 13.50 | 940 | 1.44 |
| 5363 | UvA S | Walney | 2016 | 13.50 | 780 | 1.73 |
| 5365 | UvA S | Walney | 2016 | 13.50 | 920 | 1.47 |
| 5366 | UvA S | Walney | 2016 | 13.50 | 980 | 1.38 |
| 5367 | UvA S | Walney | 2016 | 13.50 | 940 | 1.44 |
| 5368 | UvA S | Walney | 2016 | 13.50 | 895 | 1.51 |
| 5371 | UvA S | Walney | 2016 | 13.50 | 860 | 1.57 |
| 5375 | UvA S | Walney | 2016 | 13.50 | 810 | 1.67 |
| 5376 | UvA S | Walney | 2016 | 13.50 | 880 | 1.53 |
| 5377 | UvA S | Walney | 2016 | 13.50 | 880 | 1.53 |
| 5378 | UvA S | Walney | 2016 | 13.50 | 940 | 1.44 |
| 5379 | UvA S | Walney | 2016 | 13.50 | 770 | 1.75 |
| 5380 | UvA S | Walney | 2016 | 13.50 | 900 | 1.50 |
| 5381 | UvA S | Walney | 2016 | 13.50 | 940 | 1.44 |
| 5382 | UvA S | Walney | 2016 | 13.50 | 770 | 1.75 |
| 5383 | UvA S | Walney | 2016 | 13.50 | 850 | 1.59 |
| 5385 | UvA S | Walney | 2016 | 13.50 | 930 | 1.45 |
| 5386 | UvA S | Walney | 2016 | 13.50 | 810 | 1.67 |

**Colony boundaries**

Foraging trips were defined as any positional fix outside the colony area for Ribble (Fig. A1) and Walney (Fig. A2).


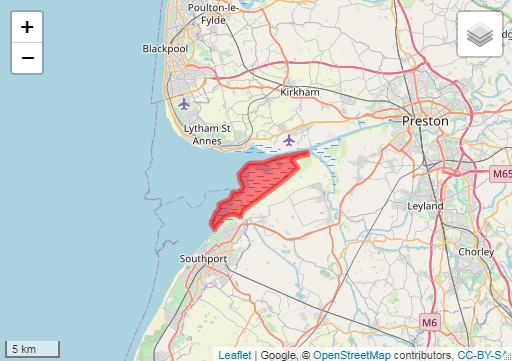


**Figure S1.** Colony area used to define foraging trips for Ribble birds (red polygon). Any GPS fixes outside the colony boundary were defined as a foraging trip


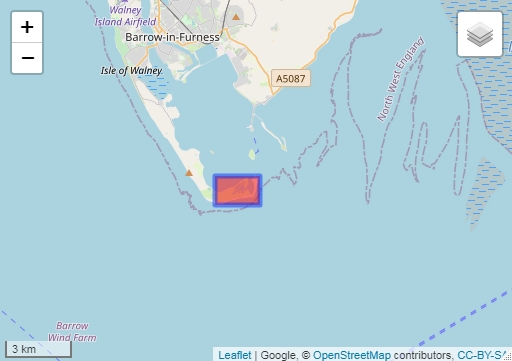


**Figure S2.** Colony area used to define foraging trips for Walney birds (blue polygon). Any GPS fixes outside the colony boundary were defined as a foraging trip

# Habitat classifications

Location points were assigned to one of seven main habitat categories (agriculture, coastal, freshwater, landfill, marine, other, urban) by grouping codes from the Corine European Landcover seamless vector database (Table S3).

**Table S3.** Habitat classifications from the Corine European Landcover database grouped into main foraging habitats types used for habitat selection modelling.abita

| **CLC Code** | **Habitat Classification** | **Main Habitat Classification** |
| --- | --- | --- |
| 111 | Continuous urban fabric | Urban |
| 112 | Discontinuous urban fabric | Urban |
| 121 | Industrial or commercial units | Urban |
| 122 | Road and rail networks and associated land | Urban |
| 123 | Port areas | Urban |
| 124 | Airports | Urban |
| 131 | Mineral extraction sites | Urban |
| 133 | Construction sites | Urban |
| 141 | Green urban area | Urban |
| 142 | Sport and leisure facilities | Urban |
| 132 | Dump | Landfill |
| 211 | Non-irrigated arable land | Agriculture |
| 212 | Permanently irrigated land | Agriculture |
| 213 | Rice fields | Agriculture |
| 221 | Vineyards | Agriculture |
| 222 | Fruit trees and berry plantations | Agriculture |
| 223 | Olive groves | Agriculture |
| 231 | Pastures | Agriculture |
| 241 | Annual crops associated with permanent crops | Agriculture |
| 242 | Complex cultivation patterns | Agriculture |
| 243 | Land principally occupied by agriculture with significant areas of natural vegetation | Agriculture |
| 331 | Beaches dunes sands | Coastal |
| 421 | Salt marshes | Coastal |
| 422 | Salines | Coastal |
| 423 | Intertidal flats | Coastal |
| 521 | Coastal lagoons | Coastal |
| 522 | Estuaries | Coastal |
| 523 | Sea and ocean | Marine |
| 411 | Inland marshes | Freshwater |
| 412 | Peat bogs | Freshwater |
| 511 | Water courses | Freshwater |
| 512 | Water bodies | Freshwater |
| 244 | Agro-forestry areas | Other |
| 311 | Broad-leaved forest | Other |
| 312 | Coniferous forest | Other |
| 313 | Mixed forest | Other |
| 321 | Natural grasslands | Other |
| 322 | Moors and heathland | Other |
| 323 | Sclerophyllous vegetation | Other |
| 324 | Traditional woodland-shrub | Other |
| 332 | Bare rocks | Other |
| 333 | Sparsely vegetated areas | Other |
| 334 | Burnt areas | Other |
| 335 | Glaciers and perpetual snow | Other |

# Habitat availability

Available habitat varied around the Ribble and Walney colonies, being dominated by agricultural and urban habitats at Ribble and agricultural and marine habitats at Walney (Fig. S3).


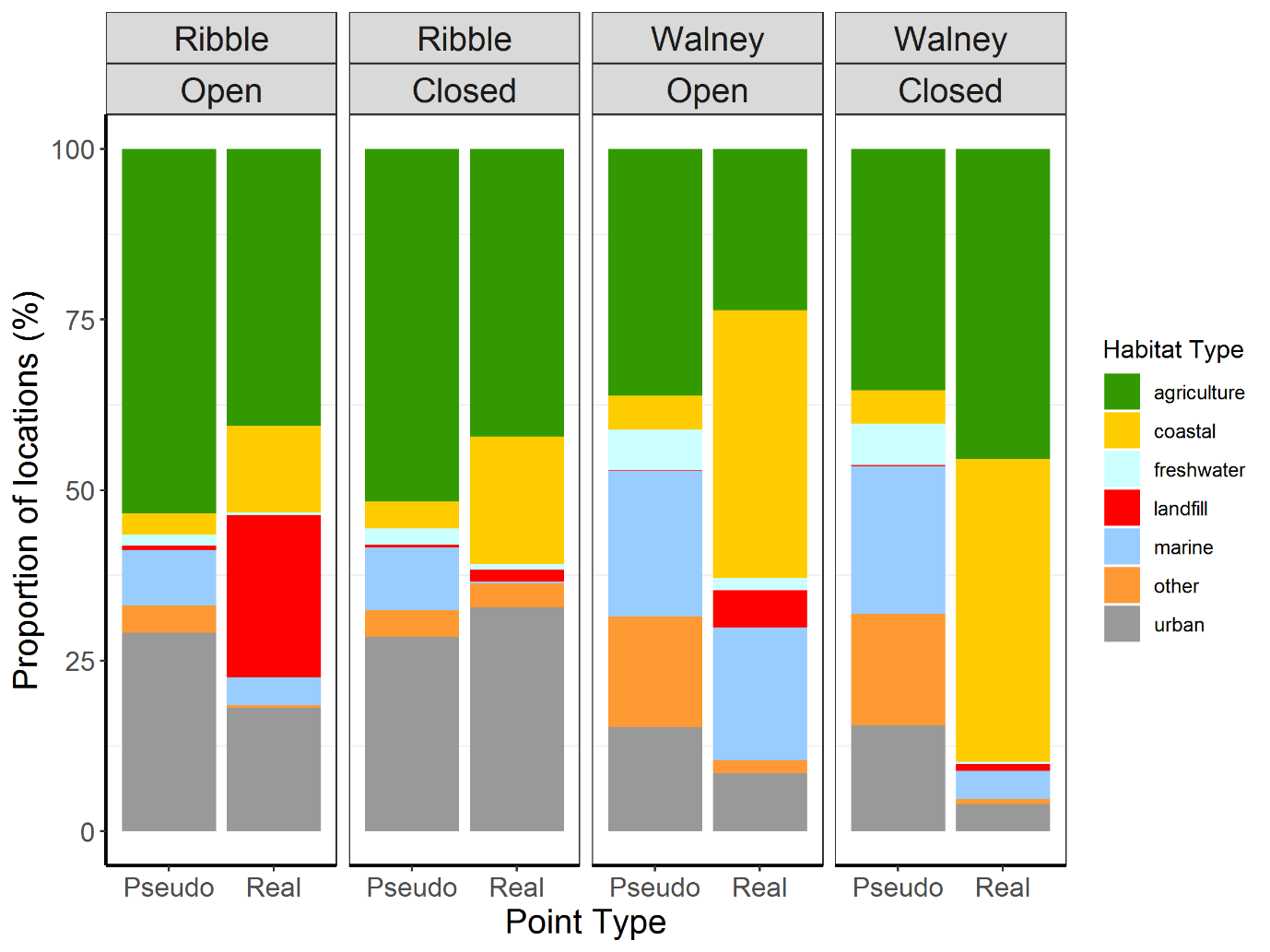


**Figure S3.** Proportion of real gull location fixes vs. randomised pseudoabsences (Pseudo) assigned to each of seven main habitat classes – agricultural, coastal, freshwater, landfill, marine, urban and other (scrub woodland and other non-foraging habitats) – for gulls breeding at Ribble and Walney in the years before and after landfill closure. Available habitat at Ribble was dominated by agricultural and urban environments whilst marine and freshwater habitats constituted a greater proportion of the pseudoabsence locations for Walney.

# Habitat Selection – Model Fit

We assessed model fit for binomial GLMMS of the probability of a location being a real gull location or a pseudo-absence by calculating the area under the receiving operator characteristic curve, predictive power, sensitivity and specificity (Table S4).

**Table S4.** Assessment of habitat selection models containing a habitat variable, site and the site*habitat interaction for all tagged birds (Table 2). All metrics are derived from a confusion matrix based on the original data. AUC (area under the receiver operating curve) ranges from 0 to 1, where 0.5 is random, and higher values indicate better model performance. CC = Correct Classification, PPP = Positive Predictive Power, NPP = Negative Predictive Power, Sen. = Sensitivity, Spec, = Specificity.

| **Model** | **Site** | **CC (%)** | **PPP (%)** | **NPP (%)** | **Sen.** | **Spec.** | **AUC** |
| --- | --- | --- | --- | --- | --- | --- | --- |
| Agriculture | Ribble | 53.09 | 19.54 | 86.16 | 0.582 | 0.521 | 0.5513 |
| Coastal | Ribble | 83.10 | 48.09 | 85.35 | 0.174 | 0.962 | 0.5682 |
| Landfill | Ribble | 84.04 | 74.75 | 84.18 | 0.064 | 0.996 | 0.5299 |
| Marine | Ribble | 24.01 | 17.87 | 97.75 | 0.990 | 0.0902 | 0.5399 |
| Urban | Ribble | 59.32 | 18.75 | 84.63 | 0.432 | 0.625 | 0.5287 |
| Agriculture | Walney | 48.95 | 19.55 | 87.07 | 0.662 | 0.455 | 0.5586 |
| Coastal | Walney | 86.07 | 62.53 | 88.95 | 0.409 | 0.951 | 0.6801 |
| Landfill | Walney | 83.89 | 85.77 | 83.87 | 0.040 | 0.999 | 0.5192 |
| Marine | Walney | 31.10 | 17.88 | 88.13 | 0.856 | 0.214 | 0.5349 |
| Urban | Walney | 28.29 | 18.01 | 91.64 | 0.930 | 0.154 | 0.5417 |

**Model Selection**

Model selection for movement data was undertaken via AIC selection using the *ICtab* function from the “bbmle” package in R. Where multiple equivalent candidate models were within ΔAIC < 2.0, the simplest model was chosen as the minimum adequate model (**Bold**; Tables S5 – S8).

**Table S5**. Top five candidate models to explain probability of visiting any landfill at the colony-level ranked by AIC weight. Pseudo-R^2^ values for the selected model - MR^2^ = 0.0399, CR^2^ = 0.328.

| **Model** | **AIC** | **dAIC** | **df** | **Weight** |
| --- | --- | --- | --- | --- |
| **Landfill ~ lf_status + (1\|ID)** | **470.2** | **0.0** | **3** | **0.49** |
| Landfill ~ lf_status + colony (1\|ID) | 471.1 | 0.9 | 4 | 0.31 |
| Landfill ~ lf_status + colony + colony*lf_status (1\|ID) | 472.0 | 1.7 | 5 | 0.20 |
| Landfill ~ 1 + (1\|ID) | 492.3 | 22.1 | 2 | <0.001 |
| Landfill ~ colony + (1\|ID) | 494.3 | 24.1 | 3 | <0.001 |

**Table S6**. Top five candidate models to explain trip duration (hrs) at the colony-level ranked by AIC weight. Pseudo-R^2^ values for the selected model - MR^2^ = 0.0833, CR^2^ = 0.299.

| **Model** | **AIC** | **dAIC** | **df** | **Weight** |
| --- | --- | --- | --- | --- |
| **Trip duration ~ lf_status + colony + (1\|ID)** | **7017.1** | **0.0** | **5** | **0.659** |
| Trip duration ~ lf_status + colony + colony*lf_status + (1\|ID) | 7018.6 | 1.5 | 6 | 0.309 |
| Trip duration ~ lf_status + (1\|ID) | 7023.1 | 6.1 | 4 | 0.032 |
| Trip duration ~ 1 + (1\|ID) | 7216.2 | 199.1 | 3 | <0.001 |
| Trip duration ~ colony + (1\|ID) | 7217.5 | 200.4 | 4 | <0.001 |

**Table S7.** Top five candidate models to explain trip length (km) at the colony-level ranked by AIC weight. Pseudo-R^2^ values for the selected model - MR^2^ = 0.067 CR^2^ = 0.271.

| **Model** | **AIC** | **dAIC** | **df** | **Weight** |
| --- | --- | --- | --- | --- |
| **Trip length ~ lf_status + colony + (1\|ID)** | **4138.2** | **0.0** | **5** | **0.46** |
| Trip length ~ lf_status + (1\|ID) | 4139.2 | 1.0 | 4 | 0.28 |
| Trip length ~ lf_status + colony + colony*lf_status (1\|ID) | 4139.3 | 1.1 | 6 | 0.27 |
| Trip length ~ colony + (1\|ID) | 4158.4 | 20.2 | 4 | <0.001 |
| Trip length ~ 1 + (1\|ID) | 4165.6 | 27.3 | 3 | <0.001 |

**Table S8.** Top five candidate models to explain distal point (km) at the colony-level ranked by AIC weight. Pseudo-R^2^ values for the selected model - MR^2^ = 0.0764, CR^2^ = 0.269.

| Model | AIC | dAIC | df | Weight |
| --- | --- | --- | --- | --- |
| **Distal point ~ lf_status + colony + colony*lf_status + (1\|ID)** | **9025.4** | **0.0** | **6** | **0.58** |
| Distal point ~ lf_status + colony + (1\|ID) | 9027.4 | 2.0 | 5 | 0.22 |
| Distal point ~ lf_status + (1\|ID) | 9027.5 | 2.1 | 4 | 0.20 |
| Distal point ~ colony + (1\|ID) | 9088.3 | 62.9 | 4 | < 0.001 |
| Distal point ~ 1 + (1\|ID) | 9094.6 | 69.1 | 3 | < 0.001 |

**Table S9:** (Generalised) Linear Mixed Model estimates ± standard error for foraging trip duration (hrs), trip length (km) and distal point distance (km) for lesser black-backed gulls in relation to landfill status and colony, with bird ID fitted as a random intercept, Estimates are from the model with the lowest AIC (see tables S6 – S8)

|  | Duration (hrs) | SE | Length (km) | SE | Max distance (km) | SE |
| --- | --- | --- | --- | --- | --- | --- |
| Intercept – landfill status open, Colony Ribble | 1.28 | 0.17 | 3.06 | 0.19 | 2.80 | 0.21 |
| Landfill status – closed | 0.72 | 0.50 | 0.45 | 0.09 | 0.26 | 0.15 |
| Colony – Walney | 0.59 | 0.20 | -0.45 | 0.22 | -0.52 | 0.23 |
| Landfill status:Colony | - | - | - | - | 0.33 | 0.17 |

# Distal points analysis

We repeated the analysis on a filtered data set containing only distal foraging trip locations, where gulls were assumed to be foraging, to account for the potential inclusion of commuting points in the main analysis. Patterns of habitat selection based on distal trip locations (Table S9; Fig. S4) were similar to those based on all foraging trip locations (Table 2; Fig. 6).

**Table S10.** Estimates for the effect of an interaction between the habitat variable and landfill status on the probability of a location being a real gull location or a pseudo-absence based on distal foraging trip locations only. Delta (Δ) AIC refers to the change in AIC caused by removing the interaction. If Δ AIC > 2, the interaction is not significant meaning we found no evidence for an effect of breeding habitat on selection for that habitat type. If the interaction effect is significant, habitat selection varied with landfill status. Models were run separately for each site. Stars next to p-values represent significance levels (* < 0.05; ** < 0.01; *** < 0.001)

| **Habitat Variable** | **Site** | **Estimate for landfill status interaction** | **p value** | **Δ AIC** |
| --- | --- | --- | --- | --- |
| Agriculture | Ribble | 0.0374 | 0.833 | -2.0 |
| Coastal | Ribble | 0.375 | 0.250 | -0.7 |
| Landfill | Ribble | -3.15 | <0.001^***^ | 18.3 |
| Marine | Ribble | -3.34 | <0.001^***^ | 47.4 |
| Urban | Ribble | 1.58 | <0.001^***^ | 49 |
| Agriculture | Walney | 1.23 | <0.001^***^ | 154.0 |
| Coastal | Walney | 0.180 | 0.300 | -0.9 |
| Landfill | Walney | -2.31 | 0.0141^*^ | 2.6 |
| Marine | Walney | -2.14 | <0.001^***^ | 225.4 |
| Urban | Walney | 0.152 | 0.325 | -1.0 |


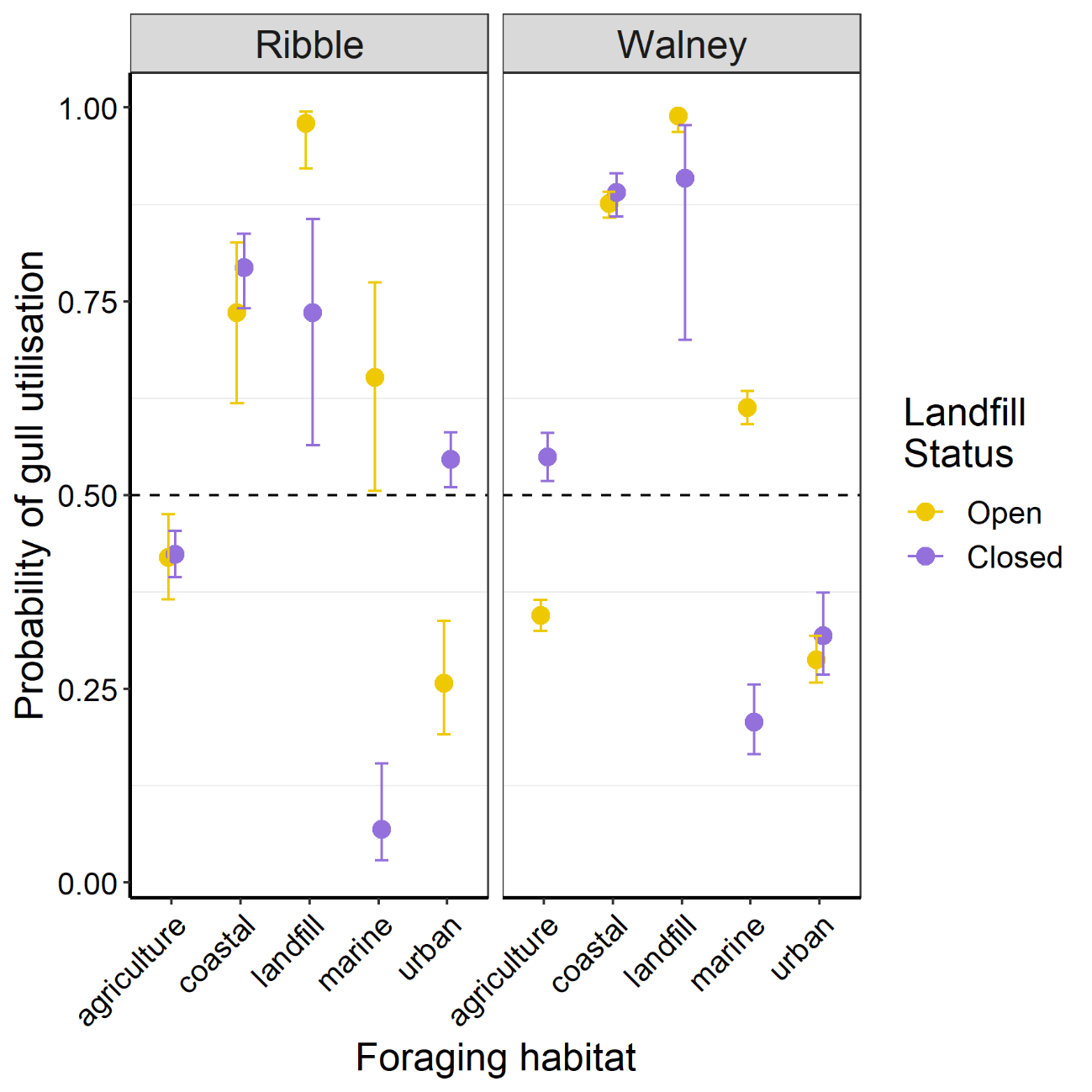


**Figure S4.** Estimates and 95% confidence intervals from resource selection models for all GPS-tagged lesser black-backed gulls breeding at Ribble and Walney before (gold) and after (purple) closure of the focal landfill site based on distal trip locations. Models estimate the probability of a given location point being a real gull location rather than a pseudo-absence in response to five main foraging habitat categories (agriculture, coastal, landfill, marine, urban). A probability of 0.50 indicates that birds used habitat in proportion to its availability whilst values of > 0.50 indicate selection for that habitat type at the colony-level.

**Table S11.** Assessment of habitat selection models for distal foraging trip locations containing a habitat variable, site and the site*habitat interaction (Table S15). All metrics are derived from a confusion matrix based on the original data. AUC (area under the receiver operating curve) ranges from 0 to 1, where 0.5 is random, and higher values indicate better model performance. CC = Correct Classification, PPP = Positive Predictive Power, NPP = Negative Predictive Power, Sen. = Sensitivity, Spec, = Specificity.

| **Model** | **Site** | **CC (%)** | **PPP (%)** | **NPP (%)** | **Sen.** | **Spec.** | **AUC** |
| --- | --- | --- | --- | --- | --- | --- | --- |
| Agriculture | Ribble | 53.73 | 20.53 | 87.23 | 0.619 | 0.521 | 0.5699 |
| Coastal | Ribble | 82.16 | 41.73 | 85.24 | 0.177 | 0.951 | 0.5639 |
| Landfill | Ribble | 84.06 | 68.57 | 84.37 | 0.0803 | 0.993 | 0.5365 |
| Marine | Ribble | 35.01 | 17.63 | 86.15 | 0.789 | 0.262 | 0.5258 |
| Urban | Ribble | 58.81 | 19.78 | 85.46 | 0.482 | 0.609 | 0.5455 |
| Agriculture | Walney | 47.87 | 20.26 | 88.65 | 0.725 | 0.429 | 0.5772 |
| Coastal | Walney | 85.16 | 59.32 | 87.98 | 0.350 | 0.952 | 0.6508 |
| Landfill | Walney | 84.27 | 92.42 | 84.18 | 0.0614 | 0.999 | 0.5302 |
| Marine | Walney | 63.04 | 21.53 | 86.02 | 0.460 | 0.665 | 0.5623 |
| Urban | Walney | 29.14 | 18.21 | 92.26 | 0.932 | 0.163 | 0.5474 |
